# Increased lesion detectability in patients with locally advanced breast cancer—A pilot study using dynamic whole-body [^18^F]FDG PET/CT

**DOI:** 10.1186/s13550-024-01096-4

**Published:** 2024-03-25

**Authors:** Mette Abildgaard Pedersen, André H. Dias, Karin Hjorthaug, Lars C. Gormsen, Joan Fledelius, Anna Lyhne Johnsson, Signe Borgquist, Trine Tramm, Ole Lajord Munk, Mikkel Holm Vendelbo

**Affiliations:** 1 Department of Nuclear Medicine & PET Centre, Aarhus University Hospital, Palle Juul-Jensens Boulevard 165, Aarhus, Denmark; 2 Department of Biomedicine, Aarhus University, Aarhus, Denmark; 3 Steno Diabetes Center Aarhus, Aarhus University Hospital, Aarhus, Denmark; 4 Department of Clinical Medicine, Aarhus University, Aarhus, Denmark; 5 Department of Radiology, Aarhus University Hospital, Aarhus, Denmark; 6 Department of Oncology, Aarhus University Hospital, Aarhus, Denmark; 7 Department of Pathology, Aarhus University Hospital, Aarhus, Denmark

**Keywords:** PET/CT, Breast cancer, Dynamic imaging, Tumor detectability

## Abstract

**Background:**

Accurate diagnosis of axillary lymph node (ALN) metastases is essential for prognosis and treatment planning in breast cancer. Evaluation of ALN is done by ultrasound, which is limited by inter-operator variability, and by sentinel lymph node biopsy and/or ALN dissection, none of which are without risks and/or long-term complications. It is known that conventional 2-deoxy-2-[^18^F]fluoro-D-glucose ([^18^F]FDG) positron emission tomography/computed tomography (PET/CT) has limited sensitivity for ALN metastases. However, a recently developed dynamic whole-body (D-WB) [^18^F]FDG PET/CT scanning protocol, allowing for imaging of tissue [^18^F]FDG metabolic rate (MR_FDG_), has been shown to have the potential to increase lesion detectability. The study purpose was to examine detectability of malignant lesions in D-WB [^18^F]FDG PET/CT compared to conventional [^18^F]FDG PET/CT.

**Results:**

This study prospectively included ten women with locally advanced breast cancer who were referred for an [^18^F]FDG PET/CT as part of their diagnostic work-up. They all underwent D-WB [^18^F]FDG PET/CT, consisting of a 6 min single bed dynamic scan over the chest region started at the time of tracer injection, a 64 min dynamic WB PET scan consisting of 16 continuous bed motion passes, and finally a contrast-enhanced CT scan, with generation of MR_FDG_ parametric images. Lesion visibility was assessed by tumor-to-background and contrast-to-noise ratios using volumes of interest isocontouring tumors with a set limit of 50% of SUVmax and background volumes placed in the vicinity of tumors. Lesion visibility was best in the MR_FDG_ images, with target-to-background values 2.28 (95% CI: 2.04–2.54) times higher than target-to-background values in SUV images, and contrast-to-noise values 1.23 (95% CI: 1.12–1.35) times higher than contrast-to-noise values in SUV images. Furthermore, five imaging experts visually assessed the images and three additional suspicious lesions were found in the MR_FDG_ images compared to SUV images; one suspicious ALN, one suspicious parasternal lymph node, and one suspicious lesion located in the pelvic bone.

**Conclusions:**

D-WB [^18^F]FDG PET/CT with MR_FDG_ images show potential for improved lesion detectability compared to conventional SUV images in locally advanced breast cancer. Further validation in larger cohorts is needed.

**Clinical trial registration:**

The trial is registered in clinicaltrials.gov, NCT05110443, https://www.clinicaltrials.gov/study/NCT05110443?term=NCT05110443&rank=1.

**Supplementary Information:**

The online version contains supplementary material available at 10.1186/s13550-024-01096-4.

## Background

Breast cancer (BC) is the most common cancer in women and the incidence has been increasing [1]. Axillary lymph node (ALN) metastasis is an important prognostic factor, and the diagnosis of ALN metastases is essential for treatment planning in patients with BC [2]. Evaluation of ALN is done clinically, by ultrasound (US), fine needle aspiration, sentinel lymph node biopsy (SLNB) and ALN dissection (ALND) [3]. As SLNB and ALND are invasive procedures they both carry risks of bleeding, infection, pain, and long-term complications [4–6]. Consequently, it is desirable to find alternative ways to examine ALN.

During a conventional 2-deoxy-2-[^18^F]fluoro-D-glucose ([^18^F]FDG) positron emission tomography / computed tomography (PET/CT), images are generated approximately one hour after tracer injection by a single whole-body (WB) pass. This results in conventional SUV images, where tissue activity is corrected for injected dose and the patient’s body weight. According to current guidelines, [^18^F]FDG PET/CT may replace traditional imaging, during initial diagnostics of BC with suspicion of metastatic disease [7]. [^18^F]FDG PET/CT has a higher sensitivity, specificity and accuracy than CT when detecting supraclavicular LN metastases [8]. Furthermore, [^18^F]FDG PET/CT can be used to guide medical decisions in case of suspicious internal mammary LN where direct biopsy is difficult, as it is more accurate than US [8]. It has also been suggested that [^18^F]FDG PET/CT staging of the axilla may be useful in some cases of non-suspicious LN on US [9]. However, conventional [^18^F]FDG PET/CT scans are suboptimal in detecting ALN metastases, with a sensitivity of 54–64% and a specificity of 89–97% [10–14]. Resolution and partial volume effects [15] are major limitations of [^18^F]FDG PET/CT as a diagnostic tool. Further limitations of the diagnostic performance of PET parameters are attributable to the relatively low [^18^F]FDG uptake by some BC, such as low grade tumors, invasive lobular carcinomas, tumors with low Ki67 index, and ER+/HER2- tumors [16], and [^18^F]FDG uptake by benign entities [15, 17, 18]. False negative findings can also be caused by micro-metastases [19].

Attempts have been made to increase the diagnostic accuracy of [^18^F]FDG PET/CT in detecting ALN metastases. Two studies examined dual-phase [^18^F]FDG PET/CT, and found that it did not improve the overall diagnostic performance for detecting ALN metastases in patients with BC [20, 21]. Time-of-flight [^18^F]FDG PET/CT has also been examined and found to have a sensitivity of 85–93% and specificity of 78–88% [19, 22]. Park et al. found that the SUVmax LN to tumor ratio was more accurate in predicting the presence of ALN metastasis than visual assessment and LN SUVmax [23].

Dynamic WB (D-WB) [^18^F]FDG PET/CT is a recently developed technique involving multiple WB passes, which, in addition to the standard SUV image, can also produce parametric images representing the net uptake rate of tracer into tissue, ${K}_{i}$ [24]. When [^18^F]FDG is used, the metabolic rate into tissue (MR_FDG_) can be found by correcting ${K}_{i}$ for the blood glucose level. Where conventional SUV images are a summation of the entire [^18^F]FDG signal, parametric images enable the distinction between free [^18^F]FDG and trapped [^18^F]FDG-6-phosphate. Several studies using dynamic [^18^F]FDG PET/CT in BC patients have been performed, and associations between kinetic parameters and tumor grade, hormone receptor status, proliferation rate, and nodal status have been reported [25–28]. However, none of the studies examined whether lesion detectability improved by the addition of parametric images, which remain a question of controversy.

Due to the risk of complications from SLNB and ALND, imaging modalities that enable more accurate classification of ALN as benign or malignant are warranted. [^18^F]FDG PET/CT is a non-invasive approach, which furthermore has the advantage of being able to evaluate both lesion morphology and glucose metabolism. Therefore, the aim of this pilot study was to examine the detectability of potential malignant lesions, and locoregional LN metastases in particular, in MR_FDG_ images compared to conventional SUV images in patients with locally advanced BC.

## Method

### Patient population

This prospective single site study included ten women with a mean age of 60 years (range: 40–84 years), all with pathological verified locally advanced BC referred consecutively to [^18^F]FDG PET/CT as part of their diagnostic work-up. All patients were scanned at Aarhus University Hospital, Denmark, in the period between April 1st 2021 and May 1st 2022. The study protocol was approved by the Central Denmark Region Committees on Health Research Ethics (1-10-72-188-19) and all participants signed an informed consent form.

### [18 F]FDG PET/CT

The [^18^F]FDG PET/CT scan was performed as part of the diagnostic process. Patients fasted for a minimum of 6 h before the administration of [^18^F]FDG (201–321 MBq). They were all scanned on a Siemens Vision 600 PET/CT in accordance with manufacturer guidelines using the fully automated multiparametric PET acquisition protocol (FlowMotion Multiparametric PET, Siemens Healthineers, Knoxville, USA). The protocol consisted of (i) a low-dose CT for attenuation correction (25 Ref mAs, 120 kV, CareDose4D, CarekV, admire level 4), (ii) a 6 min single bed dynamic scan over the chest region started at the time of tracer injection for extrapolation of an image derived input function (IDIF), (iii) a 64 min dynamic WB PET scan consisting of 16 continuous bed motion passes (7 × 2 min WB passes, followed by 9 × 5 min WB passes), and (iv) a contrast-enhanced CT scan (120 Ref mAs, 120 kV, CareDose4D, admire level 3). This protocol generated both the standard SUV image and additional parametric image. Reconstruction parameters were identical to those used by Dias et al. [24], in short SUV images were normalized to body weight and reconstructed using list-mode data (reconstruction parameters: TrueX + TOF, 4 iterations, 5 subsets, 440 matrices, 2-mm Gaussian filter and relative scatter correction). Parametric images of MR_FDG_ used the nested direct Patlak reconstruction method and were generated with list-mode data from the 6 last passes, i.e. 40–70 min, and the automatically generated IDIF (reconstruction parameters: TrueX + TOF, 8 iterations, 5 subsets, 30 nested loops, 440 matrices, 2-mm Gaussian filter and relative scatter correction).

### Kinetics

The parametric image MR_FDG_ is based on the Patlak model [29, 30]. The model assumes unidirectional net transfer of tracer, and can be used in case of irreversible uptake. In the case of [^18^F]FDG, a two-tissue compartment model with both a reversible and an irreversible compartment is assumed, where the irreversible compartment represents the phosphorylation of [^18^F]FDG to [^18^F]FDG-6-phosphate. The Patlak plot becomes linear when the reversible component is in steady-state, and the slope of the line is the rate of net influx, ${K}_{i}$. Metabolic rates were calculated as MR_FDG_ = ${K}_{i}$ ⋅ [glucose]. MR_FDG_ values were calculated for lesions with pathological uptake by indirect image-based analysis in the PKIN module of PMOD® 4.0 (PMOD Technologies Ltd, Zürich, Switzerland) using an irreversible two-tissue compartment model, the 70-min IDIF, and the 70-min activity function from the lesions contained in the bed position covering the chest, and the lumped constant set to 1 (MR_FDG_(2CM)). MR_FDG_ values were also extracted from lesions by direct reconstruction of parametric images from PET raw data (MR_FDG_(image)). Direct reconstruction was performed using the Multiparametric PET AI Suite from Siemens Healthineers on data obtained from 40 to 70 min after injection.

### Quantitative image analysis

Volume of interest (VOI) outlining was done in the PBAS module from PMOD^®^ 4.0. A VOI was placed manually covering the entire target lesion (breast tumor or metastasis) on both conventional SUV and MR_FDG_ images. Then the isocontouring tool was used for delineation with a set limit to 50% of SUV_max_ and MR_FDG_ max. Care was taken to ensure that the outlined VOI corresponded to the tumor area and was not affected by other pathology or visual artefacts. Background VOIs were placed in the immediate vicinity of target lesions as an oblong ROI in three consecutive slices. SUV_mean_ was extracted from the conventional PET reconstructions and MR_FDG_ from the parametric image. Time activity curves (TAC) were obtained from the initial 6 min scan of the chest region and the following 16 D-WB passes.

As quantitative measurements of lesion visibility, target-to-background ratio (TBR) and contrast-to-noise ratio (CNR) were used. TBR and CNR were calculated for both SUV (TBR(SUV), CNR(SUV)) and MR_FDG_ (TBR(MR_FDG_), CNR(MR_FDG_)) images, and were defined as:

$$TBR=\frac{MEAN\left(target signal\right)}{MEAN\left(background signal\right)}$$

$$CNR=\frac{MEAN\left(target signal\right)- MEAN\left(background signal\right)}{{\sigma }_{background}}$$

### Visual image analysis

An expert panel, consisting of four nuclear medicine physicians and one radiologist, all with more than 10 years of experience in PET and CT, examined the images and graded lesions. Two experts (JF and KH) graded lesions using only standard [^18^F]FDG PET/CT SUV images, while two other experts (AD and LCG) graded lesions using both standard SUV and parametric images (MR_FDG_ and DV_FDG_). Furthermore, a specialist in radiology (ALJ) inspected the contrast enhanced CT scans.

[^18^F]FDG PET/CT images (parametric and standard) were inspected using Hermes Gold Client v.2.5.0 (Hermes Medical Solution AB, Stockholm, Sweden). While Philips IntelliSpace Portal (Philips Medical Systems, Amsterdam, Netherlands) was used by the radiologist to examine CT scans. Each lesion was graded according to a certainty score of 1–5, where a score of 5 was given when the physician was confident that the lesion was malignant, 4 when the lesion was most likely malignant, 3 when it could be malignant as well as benign, 2 when it was most likely benign, and 1 when definitely benign. All scans were pseudonymized and readers were not allowed to consults others on image interpretation. Information about clinical stage and pathological results were disclosed to readers prior to inspection of the scans.

### Statistical analysis

The normality of the data was checked using QQ-plots. MR_FDG_ values are presented as means with associated 95% confidence intervals (95% CI). MR_FDG_ values from tumors were compared to MR_FDG_ values in glandular breast tissue by a paired t-test. Correlation between MR_FDG_(2CM) and MR_FDG_(image) was examined by simple linear regression and Bland-Altman plot was used for visual evaluation of agreement. TBR(MR_FDG_) values were compared with TBR(SUV) values, and CNR(MR_FDG_) values with CNR(SUV) values, using log-transformation and paired t-test, results were given as median ratios with 95% CI. Interrater agreement examined the physicians’ certainty score agreement, by the use of intraclass correlation (ICC) estimates and their 95% confidence intervals (CI). ICC was based on absolute-agreement and used a two-way random-effect model [31]. ICC values < 0.5 indicate poor agreement, 0.5–0.75 moderate agreement, 0.75–0.9 good agreement, and > 0.9 excellent agreement [32]. All statistics were calculated using STATA version 17.0 (StataCorp. 2021. Stata Statistical Software: Release 17. College Station, TX: StataCorp LLC).

## Results

### Baseline

Ten women with BC were included, all referred to a [^18^F]FDG PET/CT scan for the purpose of staging. Eight patients had newly diagnosed locally advanced BC, defined as clinical stage IIIA-C [33], and two had locally advanced recurrence of BC, clinical stage IIIA-C. For pathological details, see Table 1, and for detailed TNM classification and stage, see supplemental Table S1. They were all scanned with the D-WB PET protocol. Their mean age was 60.1 years (range 40–84 years). Lesions with pathological verification consisted of 11 breast tumors, 11 axillary LN metastases/satellite tumors, and four supraclavicular LN metastases/satellite tumors. Lesions deemed malignant on [^18^F]FDG PET/CT constituted 13 breast tumors, 39 ALN metastases, 20 other LN metastases, and 13 bone metastases.

**Table 1:** Pathology

Ductal/lobular (n)	8/2
ER+/HER2 2+ (n)	5
ER-/HER2 2+ (n)	0
ER+/HER2-	4
ER-/HER2- (triple neg.)	1
Grade 1/2/3 (n)	1/5/4

Pathology results for the ten included patients

### Kinetics

**Fig. 1:**
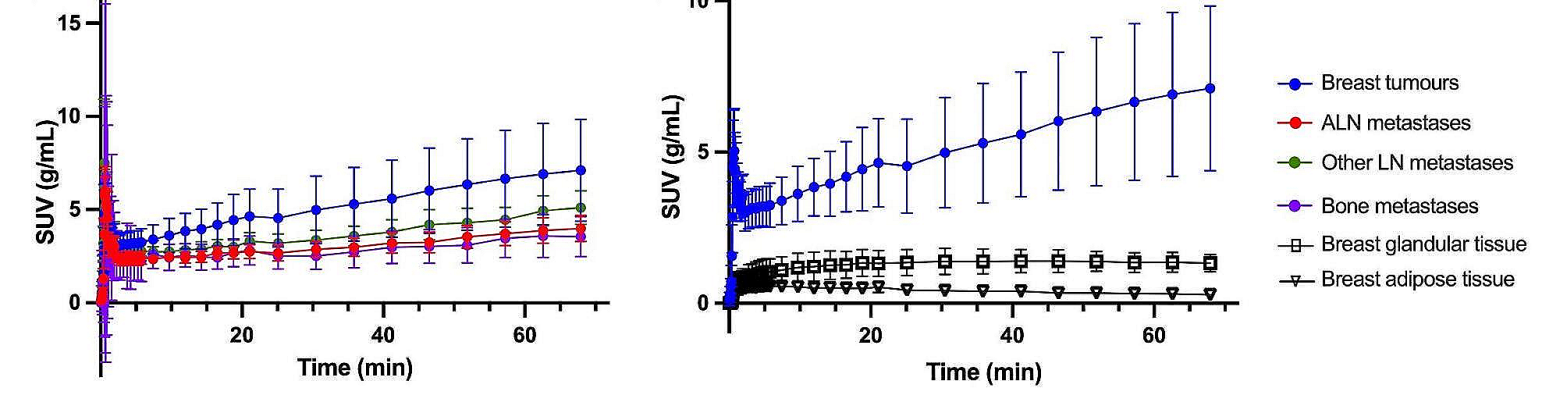
Time activity curves from tumor lesions and breast tissue, presented as mean values (standard deviation) of all lesions/tissues from all patients. **a**) Time activity curves of breast tumors and metastases, **b**) Time activity curves in breast tumors, breast glandular tissue, and breast adipose tissue

TAC for breast tumors and metastases are shown in Fig. 1a. Within the first two min after [^18^F]FDG injection, there was an activity peak, followed by a swift decrease. From two min and onwards there was a continuous increase. The activity patterns were similar for breast tumors and metastases, although breast tumors tended to have a higher SUV_mean_. In Fig. 1b it is evident that the activity level in breast tumors was significantly higher compared to background activity in the glandular and adipose tissue of the breast.

**Fig. 2:**
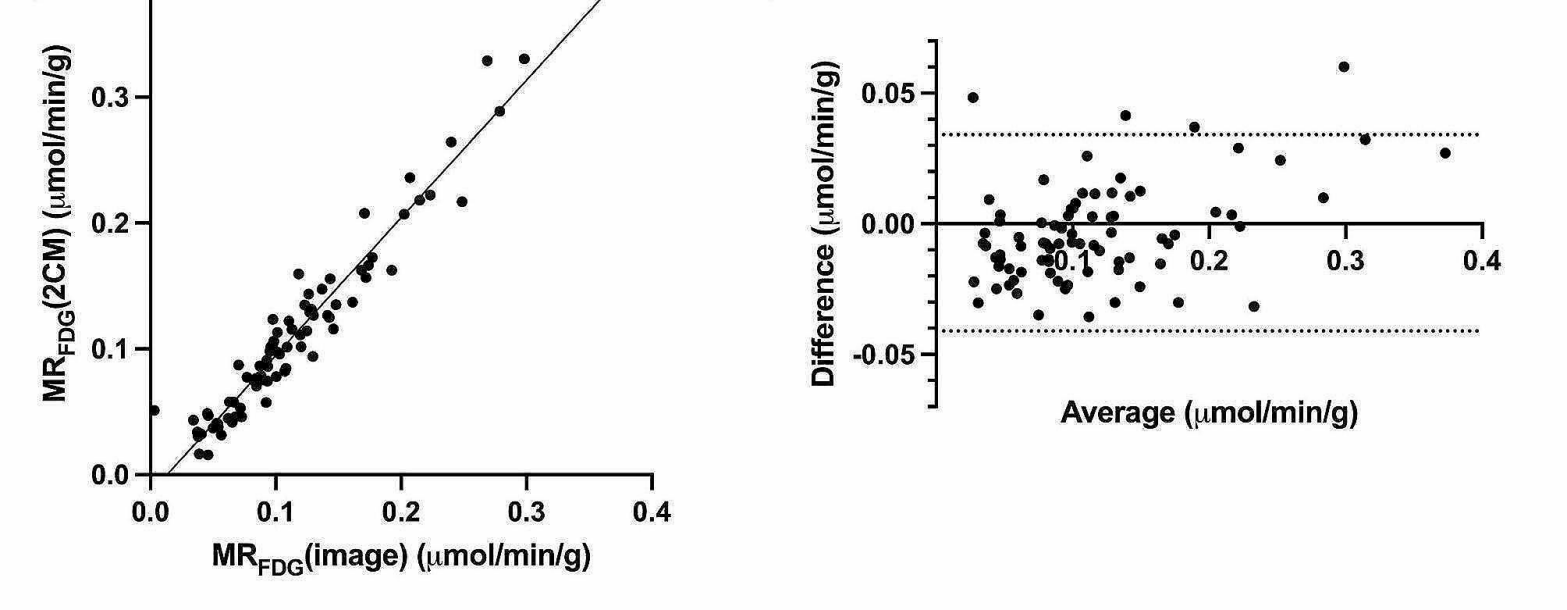
**a**) Correlation of MR_FDG_(image) values from direct reconstruction of parametric images from PET raw data and MR_FDG_(2CM) from indirect image-based analysis. **b**) Bland-Altman analysis of difference vs. average

The mean MR_FDG_(image) was 0.17 µmol/min/g (95% CI: 0.11–0.23) for breast tumors, 0.11 µmol/min/g (95% CI: 0.10–0.12) for LN metastases, 0.02 µmol/min/g (95% CI: 0.01–0.02) for normal glandular breast tissue, and 0.002 µmol/min/g (95% CI: 0.001–0.003) for normal adipose breast tissue. There was a statistically significant difference in MR_FDG_(image) between tumor tissue and glandular breast tissue, *p* < 0.001. When comparing MR_FDG_ (image) and MR_FDG_ (2CM), we found an excellent correlation, R^2^ = 0.94 (Fig. 2).

### Quantitative measurement of lesion visibility

**Fig. 3:**
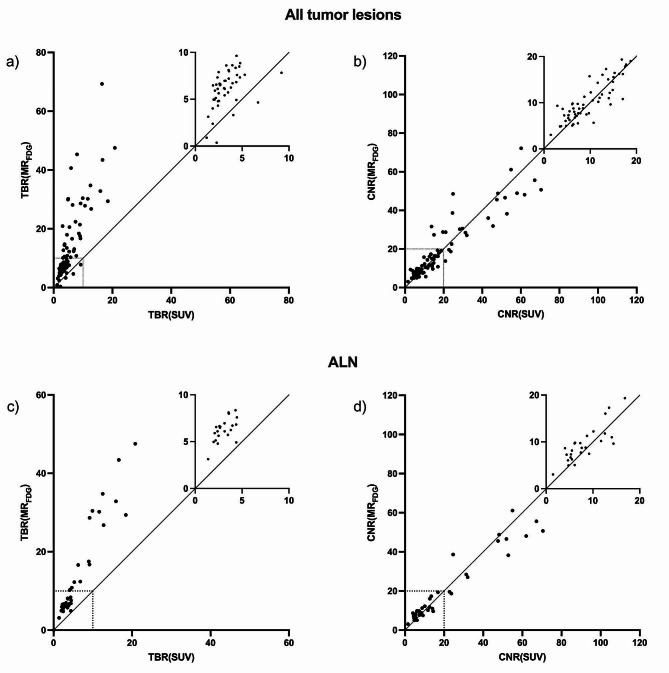
Quantitative lesion visibility **a**) TBR(SUV) vs. TBR(MR_FDG_) for all lesions in all patients, **b**) CNR(SUV) vs. CNR(MR_FDG_) for all lesions in all patients, **c**) TBR(SUV) vs. TBR(MR_FDG_) for ALN metastases, **d**) CNR(SUV) vs. CNR(MR_FDG_) for ALN metastases

TBR(MR_FDG_) was significantly higher than TBR(SUV), *p* < 0.001, with TBR(MR_FDG_) values being 2.25 (95% CI: 1.95–2.52) times higher than the TBR(SUV) values. CNR(MR_FDG_) was 1.08 (95% CI: 1.01–1.16) times higher than CNR in SUV images, *p* = 0.02 (Fig. 3). This indicated that lesion detectability was better on the MR_FDG_ images. TBR(MR_FDG_) values were higher than TBR(SUV) values in 80 lesions out of 85 (94%), while 49 out of 85 (58%) had a higher CNR(MR_FDG_) compared to CNR(SUV). The lesions with low TBR(MR_FDG_) and CNR(MR_FDG_) included all types of tumors; breast tumors, LN metastases, and bone metastases.

### Axillary lymph nodes

The same pattern was evident when examining ALN metastases exclusively, here TBR(MR_FDG_) was 2.19 (95% CI: 2.04–2.35) times higher than TBR(SUV), *p* < 0.001 (Fig. 3). Notably, TBR(MR_FDG_) was higher than TBR(SUV) in all ALN metastases.

### Visual lesion detectability

**Fig. 4:**
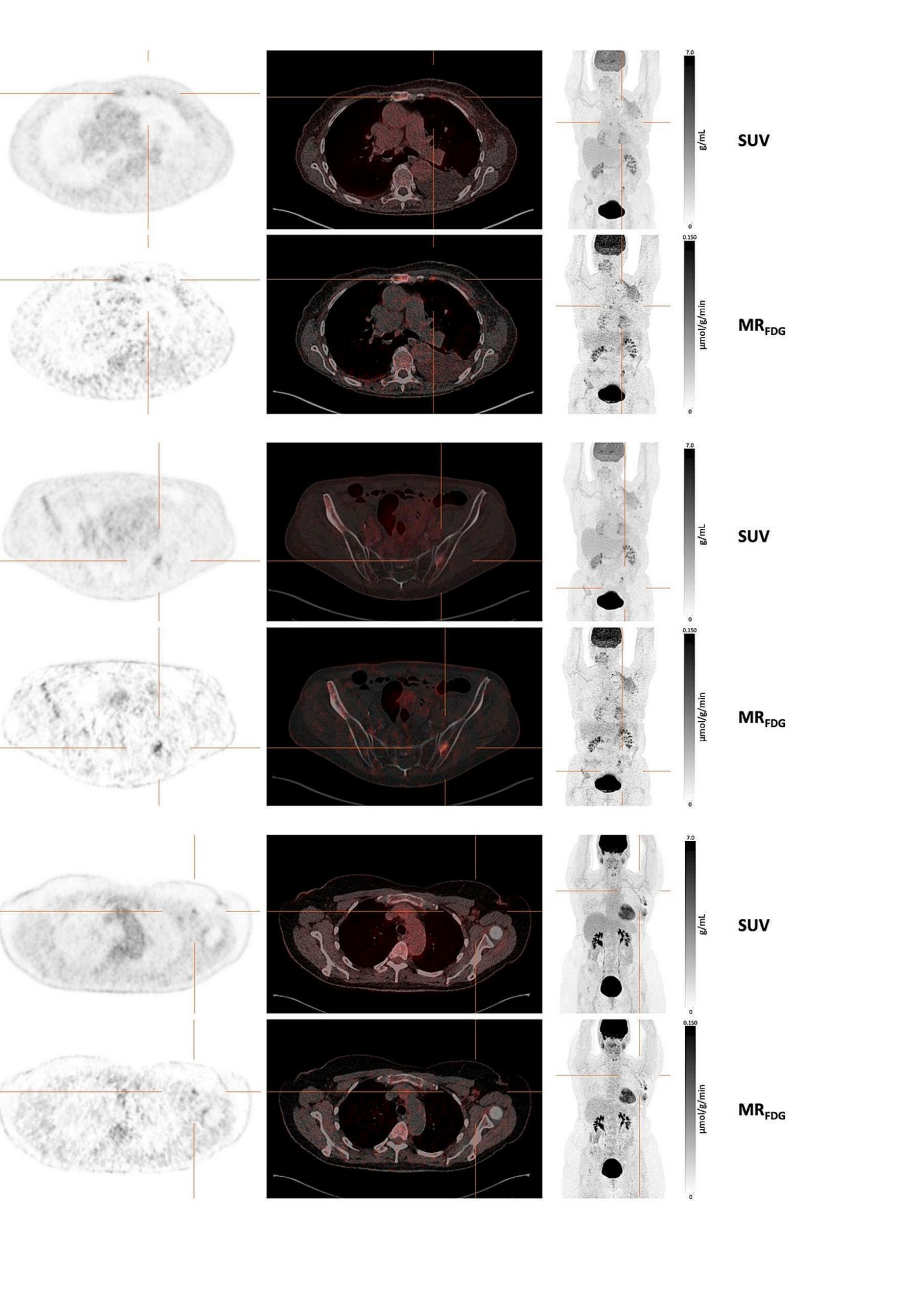
Additional lesions detected on MR_FDG_ images. **a**) Parasternal LN, **b**) Bone lesion in the left ilium in the same patient as **a**), **c**) LN lesion in level I of the left axilla

There was a moderate interrater agreement in both expert groups. ICC was 0.65 (95% CI: 0.47–0.77) among experts who assessed SUV images and 0.63 (95% CI: 0.44–0.75) among experts who assessed both SUV and MR_FDG_ images (see Table 2 for individual scores). A total of three extra lesions in two patients were detected when both standard SUV and MR_FDG_ images were available (Fig. 4). The three extra lesions detected received different certainty scores. One lesion in the left axillary had a score of four, meaning it was most probable malignant. Two lesions, one parasternal LN and one in the pelvic bone, had certainty scores of five, meaning they were estimated to be malignant. There was consensus among the readers: none of the lesions were identified by those reviewing SUV images, yet both readers examining the MR_FDG_ images classified them as most likely malignant or malignant. Compared to the time of referral, the conventional SUV images increased the disease stage in three patients (Table S1). However, there was no change in disease stage due to the detection of extra lesions in M_RFDG_ images, and none of these lesions were pathologically verified.

**Table 2:**
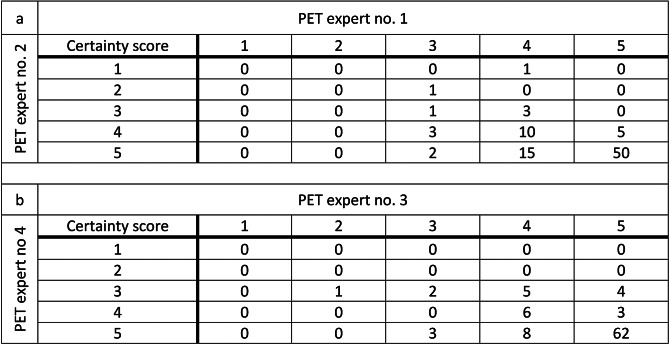
Certainty scores

Individual certainty scores from the four PET experts. PET expert no. 1 and 2 assessed SUV images exclusively. PET expert no. 3 and 4 assessed both SUV and MR_FDG_ images

The radiology expert who exclusively reviewed the contrast-enhanced CT scans identified fewer lesions in six out of ten scans when compared to the findings from the [^18^F]FDG PET/CT scans. In two patients, lesions that appeared as a single large lesion on [^18^F]FDG PET/CT could be distinguished as two and three separate and smaller lesions on the CT scans.

## Discussion

This is the first study to examine the diagnostics of D-WB [^18^F]FDG PET/CT compared to standard WB [^18^F]FDG PET/CT for the identification of BC lesions, including ALN metastases. The quantitative measures of tumor visibility were consistently higher for TBR(MR_FDG_) and mostly higher for CNR(MR_FDG_). This resulted in the detection of three additional suspicious lesions in two out of ten patients when experts examined MR_FDG_ images in addition to the conventional SUV images.

We found an excellent correlation between MR_FDG_(image) values extracted directly from the image reconstruction generated from PET raw data and MR_FDG_(2CM) values from the full kinetic analyses made in PMOD® 4.0. These findings suggest that the MR_FDG_(image) values match and can replace the more comprehensive image-based MR_FDG_(2CM) calculation, which is in alignment with previous findings [34]. The mean MR_FDG_ values for breast tumors in this study agree with prior results where a significant difference between breast tumors and breast tissue was also observed [25, 27].

As a consequence of the low sensitivity in detecting ALN metastases, [^18^F]FDG PET/CT scans are primarily used to identify distant metastases. We aimed to evaluate any added value of MR_FDG_ images in the detection of tumor lesions, with a particular emphasis on axillary and parasternal LN metastases, in patients with locally advanced BC. Conventional SUV images are used in the initial staging of numerous cancer types [35]. In recent years, new scanning techniques have enabled the production of D-WB PET/CT scans feasible in a clinical setting and automated reconstruction algorithms can easily produce MR_FDG_ images from PET raw data [24]. Furthermore, with the recent introduction of total-body PET scanners, better dynamic images with reduced noise are now a possibility. To quantitatively evaluate the added value of MR_FDG_ images, TBR and CNR were examined. For this, SUVmean from a VOI outlined by a set limit of 50% of maximum was utilized instead of alternative SUV metrics such as SUV_max_ or SUV_peak_. This method was selected to maintain consistency with prior related studies and to adhere to established guidelines for defining tumor VOIs [24, 36]. We found TBR(MR_FDG_) to be highest in 80 lesions out of 85 (94%), as a result of the reduced signal from the background. This is in agreement with a study examining 18 patients referred for staging or restaging of abdominal or lung lesions by [^18^F]FDG PET/CT [37]. Likewise, it has been reported that TBR in MR_FDG_ images is superior to TBR in SUV images in 299/310 lesions (96%) [24]. CNR, which also account for background noise, was highest in MR_FDG_ images in 49 of 85 lesions (58%). This indicated that, in just about 60% of cases MR_FDG_ images have the potential to aid in lesion detection. A proportion that others have reported to be even greater [37].

In the visual assessment of images there was moderate agreement between the two experts assessing the conventional SUV images and the experts who, in addition, also assessed MR_FDG_ images. This is similar to previously reported interrater agreements in [^18^F]FDG PET/CT assessments in patients with BC and other cancer types [38, 39]. In Prior studies, examining the potential clinical benefits gained by addition of parametric images Dias et al. examined the visual detectability of pathological lesions in MR_FDG_ compared to standard SUV images in a mixed cohort of 101 patients and found no additional lesions on MR_FDG_ images [24]. In a later study, normal values for [^18^F]FDG uptake in selected tissues were estimated and no difference was observed between malignant and non-malignant [^18^F]FDG avid lesions in MR_FDG_ images [34]. In contrast to this, a study examining 135 LN in 29 patients with lung cancer found that ${K}_{i}$ values could discriminate malignant from benign LN [40], while an additional, pathological verified, liver lesion was detected in a population of 18 oncological patients [37]. In the present study, the assessment of MR_FDG_ images resulted in the identification of two extra potential locoregional LN metastasis, one in the axillary level I and one located parasternal. This highlights the potential for increased sensitivity in the detection of LN metastases through the utilization of parametric imaging. However, the possibility of eliminating SLNB and ALND remains distant due to the possible presence of micrometastases, sometimes limited [^18^F]FDG uptake in BC, and the spatial resolution limits of both traditional and parametric PET/CT scans. Nonetheless, the use of more cancer specific tracers, such as [68Ga]FAPI, which has demonstrated encouraging outcomes in BC [41], could enhance the detectability of lesions to some extent. MR_FDG_ images also revealed an additional bone metastasis. The three lesions were all assigned certainty scores of four or five, meaning the expert readers deemed it malignant or most probable malignant. Nevertheless, none of the additional findings led to an alteration in the disease stage, as there was already evidence of metastasis at the same axillary level or distant metastasis observed in conventional SUV images. It has been reported that parametric imaging can reduce the number of false-positive findings [24, 37]. We were unable to investigate this, as all lesions seen on SUV images were also present on MR_FDG_ images. However, as conventional [^18^F]FDG PET/CT already have a high specificity in BC patients, it is unlikely that this will, in itself, lead to alterations in recommendations.

In line with current knowledge, conventional SUV images were superior to contrast-enhanced CT scans in the detection of tumors [8]. However, in one area, the lungs, CT scans are known to have a higher sensitivity [42]. None of the included patients had any visual lung metastases, hence we were unable to evaluate detection of lung abnormalities. In two patients, the heightened resolution of the contrast-enhanced CT scans, resulted in the differentiation of what appeared to be a single large lesion on [^18^F]FDG PET/CT into two and three smaller separate lesions.

This study has limitations. First, being a pilot study, our population was small, comprising only ten patients, and heterogeneous, with both newly diagnosed and recurrent BC. However, the enrolled patients were known to have clinical advanced disease and as such a relatively large number of relevant lesions were present for the analysis. We were unable to match LN evaluated by [^18^F]FDG PET/CT with those that underwent pathologic examination in a one-to-one manner. However, comparison of final stage from [^18^F]FDG PET/CT results matched the results from the combined diagnostic pathology and imaging reports. As a major limitation, none of the three additional lesions detected on MR_FDG_ images were referred for pathological verification, as this would have had no effect on disease stage. The prolonged scan protocol did not result in motion artifacts. Despite this, an imaging protocol of 70 min will most likely lead to increased patient discomfort with possible motion artifacts as a result. Furthermore, an imaging protocol of 70 min will be incompatible with the increasing demand for PET/CT scans. As a consequence, a reduction in scan time is crucial and it has been proven possible to reduce the D-WB parametric imaging protocols to 20 min by using a population-based input function [43].

## Conclusions

In conclusion, this study underlines the potential of increased diagnostic accuracy using D-WB [^18^F]FDG PET/CT compared to conventional WB [^18^F]FDG PET/CT, resulting from parametric imaging conducted as an addition to conventional SUV images. These results need to be validated prospectively in a larger cohort where pathological verification is essential.

### Electronic supplementary material

Below is the link to the electronic supplementary material.

Supplementary Material 1

## Data Availability

The data used in the current study is available from the corresponding author on reasonable request.

## Abbreviations

BC: Breast cancer
ALN: Axillary lymph node
US: Ultrasound
SLNB: Sentinel lymph node biopsy
ALND: Axillary lymph node dissection
[^18^F]FDG: 2-deoxy-2-[^18^F]fluoro-D-glucose
PET/CT: Positron emission tomography / computed tomography
D-WB: Dynamic whole-body
VOI: Volume of interest
TAC: Time activity curves
TBR: Target-to-background ratio
CNR: Contrast-to-noise ratio
ICC: Intraclass correlation
CI: Confidence intervals
