# Supplementary material for: Increased lesion detectability in patients with locally advanced breast cancer—A pilot study using dynamic whole-body [18F]FDG PET/CT
Source: EJNMMI Res. 2024 Mar 25;14:31. doi: 10.1186/s13550-024-01096-4 (PMC10963357; doi:10.1186/s13550-024-01096-4)
Supplement: Supplementary file 1 — Supplementary Material 1 [file 13550_2024_1096_MOESM1_ESM.docx]

# Increased lesion detectability in patients with locally advanced breast cancer – A pilot study using dynamic whole-body [^18^F]FDG PET/CT

Mette Abildgaard Pedersen^1,2,3^, André H. Dias^1^, Karin Hjorthaug^1^, Lars C. Gormsen^1,4^, Joan Fledelius^1^, Anna Lyhne Johnsson^5^, Signe Borgquist^4,6^, Trine Tramm^4,7^, Ole Lajord Munk^1,4^, Mikkel Holm Vendelbo^1,2,3^

^1^ Department of Nuclear Medicine & PET Centre, Aarhus University Hospital, Aarhus, Denmark.

^2^ Department of Biomedicine, Aarhus University, Aarhus, Denmark

^3^ Steno Diabetes Center Aarhus, Aarhus University Hospital, Aarhus, Denmark

^4^ Department of Clinical Medicine, Aarhus University, Aarhus, Denmark

^5^ Department of Radiology, Aarhus University Hospital, Aarhus, Denmark

^6^ Department of Oncology, Aarhus University Hospital, Aarhus, Denmark

^7^ Department of Pathology, Aarhus University Hospital, Aarhus, Denmark

Supplemental material

| **Table S1: TNM and disease stage** | | | | | |
| --- | --- | --- | --- | --- | --- |
| Referral | | SUV images | | SUV and MR_FDG_ images | |
| TNM | Stage | TNM | Stage | TNM | Stage |
| T2N3M0 | IIIC | - | - | - | - |
| T3N2M0 | IIIA | T3N2M1 | IV | -- | -- |
| T3N1M0 | IIIA | T2N1M0 | IIB | -- | -- |
| T1N2M0 | IIIA | T2N3M1 | IV | -- | -- |
| T3N1M0 | IIIA | - | - | - | - |
| T4N3M0 | IIIC | T4N3M1 | IV | -- | -- |
| T2N3M0 | IIIC | - | - | - | - |
| T3N1M0 | IIIA | T2N1M0 | IIB | -- | -- |
| T0N3M0 | IIIC | - | - | - | - |
| T3N2M0 | IIIA | - | - | - | - |

| **Table S1: TNM and disease stage** | | | | | |
| --- | --- | --- | --- | --- | --- |
| Referral | | SUV images | | SUV and MR_FDG_ images | |
| TNM | Stage | TNM | Stage | TNM | Stage |
| T2N3M0 | IIIC | - | - | - | - |
| T3N2M0 | IIIA | T3N2M1 | IV | -- | -- |
| T3N1M0 | IIIA | T2N1M0 | IIB | -- | -- |
| T1N2M0 | IIIA | T2N3M1 | IV | -- | -- |
| T3N1M0 | IIIA | - | - | - | - |
| T4N3M0 | IIIC | T4N3M1 | IV | -- | -- |
| T2N3M0 | IIIC | - | - | - | - |
| T3N1M0 | IIIA | T2N1M0 | IIB | -- | -- |
| T0N3M0 | IIIC | - | - | - | - |
| T3N2M0 | IIIA | - | - | - | - |

Table S1: TNM classification and disease stage for the individual patients upon referral, according to conventional SUV images, and according to SUV and additional MR_FDG_ images. In five out of ten patients, the conventional SUV images caused a change in disease stage. In none of the ten patients the MR_FDG_ images caused a change in disease stage when compared to conventional SUV images. - represents unchanged TNM classification and disease stage compared to upon referral. -- represents unchanged TNM classification and disease stage compared to SUV images.
